# Retraction: Measurement and analysis of the distortion of factor prices in China

**DOI:** 10.1371/journal.pone.0335970

**Published:** 2025-11-04

**Authors:** The PLOS One Editors

After this article [1] was published, concerns were raised about potential manipulation of the publication process. Specifically:

- The study reported in [1] appears similar to previously published work [2] (citation #44 of [1]); datasets (both data sources and timeframe of data collection), variables and methodology reported in [1], appear similar to [2], although the reported quantitative data are different.
- The articles cited as References 2, 11–16, and 35 in [1] appear to be irrelevant to this article [1].
- There are errors in the DOIs for References 17–19 of [1], and Reference 25 of [1] is a duplicate of Reference 20 of [1].
- The *PLOS One* Editors identified concerns about the article’s peer review.

PLOS consulted a member of the *PLOS One* Editorial Board who advised that the contribution of [1] compared with [2] is insufficient to warrant a separate publication and the single reference to [2] (citation #44 in [1]) is insufficient to place the work in context. They also stated that the above references of concern (citations #2, 11–16, and 35 of [1]) did not appear to support the corresponding text.

The consulting Editorial Board Member also advised that there are issues in the reporting of the methodology used in [1]. Specifically, they noted that there is a lack of detail in the description of the panel data and how to access the data source, and that the limitations of the regression models used and the observed results require further discussion in relation to the statistical methodologies employed.

The authors of [1] did not respond to any communications from PLOS regarding the similarity with [2] or follow-up queries.

In light of the above cumulative unresolved concerns, the *PLOS One* Editors retract this article. We regret that the issues were not identified prior to the article’s publication.

All authors did not agree with the retraction.
